# Lehrbuch Der Hebammenkunst

**Published:** 1892-06

**Authors:** 


LEHRBUCH DER HEBAMMENKUNST. Von DR. BERNHARD SIGMUND SCHULTZE, Geheim Hofrath, Öff. Ord.; Prof. der Geburtshülfe; Director der Entbindungsanstalt und der Hebammenschule zu Jana; Mitglied der Medical Commission des Grossherzogth. Sachsen. Leipzig: Verlag von Wilhelm Engelmann. 1891. Pp. 380. Zehnte Auflage.

The ninth edition of this work was reviewed in the April (1890) number of the JOURNAL, p. 588. In speaking of the ninth edition, we ventured the assertion that it "would be as enthusiastically received as the former editions." Scarcely eighteen months had elapsed, and the edition was exhausted, necessitating the publishing of a tenth edition. This fact speaks of its popularity and value more than a reviewer's pen can hope to do, and consequently it is laid one side. No changes whatever have been made in this edition. W. C. K.

